# A study on the relationship between digital leadership, digital self-efficacy, and work engagement: the moderating role of technostress

**DOI:** 10.3389/fpsyg.2026.1751207

**Published:** 2026-02-12

**Authors:** Jingke Li, Yongkang Wang, Joongoo Han

**Affiliations:** 1 Graduate School, Kangnam University, Yongin, Republic of Korea; 2 Division of Global Business Administration, Kangnam University, Yongin, Republic of Korea

**Keywords:** conservation of resources theory, digital leadership, digital self-efficacy, technostress, work engagement

## Abstract

**Introduction:**

With the rapid development of emerging technologies, organizations are experiencing a profound digital transformation. However, in this high-pressure context, employees frequently experience technostress, which may diminish the positive influence of leadership. Drawing on Conservation of Resources (COR) theory, this study constructs a mediation model linking digital leadership, digital self-efficacy, and work engagement, while incorporating technostress as a moderating variable.

**Methods:**

Using a two-wave time-lagged design, this study collected survey data from 352 employees across multiple companies in China.

**Results:**

(1) digital leadership significantly enhances employee work engagement, (2) digital self-efficacy partially mediates this association, and (3) the positive influence of digital leadership on digital self-efficacy is substantially weakened under high technostress.

**Discussion:**

These findings extend the applicability of COR theory to digital contexts and highlight the boundary role of technostress.

## 1 Introduction

Since the beginning of the 21st century, the rapid development of emerging technologies including big data, artificial intelligence, cloud computing, and the Internet of Things has driven organizations worldwide to undergo an unprecedented digital transformation. Digital transformation is not simply a technological upgrade but a systemic process that fundamentally reshapes organizational logic, employee work practices, and strategic directions (Bharadwaj et al., 2013). As Hanelt et al. (2021) observed, digital transformation has become a decisive factor for firms seeking to remain competitive in turbulent markets.

During this process, the role of organizational leaders has undergone a fundamental transformation. Traditional leadership depends largely on experience and interpersonal interaction, whereas digital transformation requires managers to demonstrate digital leadership, defined as the ability to understand and use digital technologies to achieve strategic goals (Zeike et al., 2019). Consequently, scholars increasingly recognize digital leadership as a critical driving force behind successful organizational transformation.

However, the advancement of digital transformation entails certain costs. Numerous studies show that as organizations adopt digital tools extensively, employees inevitably experience technostress (Tarafdar et al., 2007). Technostress describes the psychological and physiological strain that employees experience while adapting to rapidly evolving information technologies, which may lead to anxiety, fatigue, or burnout (Ayyagari et al., 2011). Arnetz and Wiholm (1997) were among the first to identify technostress, arising from information overload and system complexity, as a direct threat to employee health and performance. More recent studies have further demonstrated the increasing prevalence of technostress. Marsh et al. (2022) found that in highly digitalized work environments, although employees experience higher efficiency, they also report greater stress due to increased information-processing demands and task complexity. A meta-analysis by (Gilboa et al., 2008) revealed a significant negative association between technostress, employee performance, and innovative behavior.

Among the psychological and behavioral outcomes affected by technostress, work engagement, a key indicator of employees’ positive psychological state, merits particular attention. Work engagement is defined as the vigor, dedication, and absorption that employees demonstrate in their work (Schaufeli and Bakker, 2004). Studies indicate that excessive technostress significantly diminishes employees’ work engagement (Bondanini et al., 2020). At the same time, digital leadership strengthens employees’ identification with organizational goals, thereby enhancing their work engagement (Zeike et al., 2019). Li et al. (2024) found that when leaders provide employees with digital empowerment and supportive resources, they display higher levels of engagement and creativity. Emphasized that digital leadership reduces employees’ uncertainty and enhances psychological safety in digital contexts, thereby improving work engagement.

In digital environments, digital self-efficacy is regarded as a crucial psychological resource that enables employees to manage technological challenges effectively (Compeau and Higgins, 1995). Research demonstrates that digital leadership effectively enhances employees’ digital self-efficacy through training and support (Sun and Yoon, 2025). Moreover, digital self-efficacy enhances employees’ work engagement by reducing uncertainty and anxiety and by increasing their sense of control over tasks (Salanova et al., 2002). Therefore, digital self-efficacy plays a crucial mediating role in linking digital leadership to work engagement. In other words, digital leaders indirectly foster employees’ work engagement by reinforcing their digital self-efficacy.

Overall, although prior research underscores the positive effects of digital leadership on work engagement, most studies have overlooked technostress as a critical contextual factor in digital transformation. Technostress may modify or weaken the mechanism through which digital leadership exerts its effects. Moreover, existing studies on digital leadership and technostress have been conducted predominantly in Western organizational contexts. China, as an emerging economy undergoing rapid digital transformation and characterized by distinct cultural and institutional features, provides a relevant empirical setting in which the proposed relationships can be examined. Furthermore, while the positive role of digital self-efficacy has been confirmed, its mediating function in the relationship between digital leadership and work engagement remains underexplored. Therefore, this study develops a mediation model with digital self-efficacy as the mediator to examine how digital leadership affects work engagement and introduces technostress as a moderator to explore whether digital leadership can enhance employee engagement under conditions of digital transformation and high technostress.

## 2 Hypothesis and hypotheses

### 2.1 Technostress

Technostress, a stressor closely related to information and communication technology (ICT), was first introduced by Brod (1984) in Technostress: The Human Cost of the Computer Revolution. He defined it as “a modern adaptive disorder resulting from an inability to cope effectively with new computer technologies.” As information technology continues to advance, scholars have gradually broadened and deepened their understanding of technostress. Arnetz and Wiholm (1997) described it as “psychophysiological symptoms shown by employees who are highly dependent on computers in their work,” highlighting its negative effects on physical and mental health. Similarly, Weil and Rosen (1997) broadened the concept, defining technostress as “any negative impact that technology has, directly or indirectly, on attitudes, thoughts, behaviors, or the body,” thereby extending its conceptual scope.

In the 21st century, research on technostress has increasingly focused on organizational contexts. Tarafdar et al. (2007) developed the Technostress Creators Scale, identifying five dimensions: techno-overload, techno-invasion, techno-complexity, techno-insecurity, and techno-uncertainty. They demonstrated that these factors significantly affect employees’ job satisfaction and performance. Building on this work, Ragu-Nathan et al. (2008) identified three key characteristics of modern workplace technology: increasing organizational dependence on ICT, widening technological divides, and the transformation of traditional work environments and cultures.

Recent research has increasingly highlighted the “dark side” of technostress. Marsh et al. (2022) observed that the proliferation of email and smartphones keeps employees constantly connected, creating an “always-on” work condition. Ayyagari et al. (2011) found that technostress originates not only from the characteristics of technology itself, such as complexity and excessive use, but also from task demands and individual perceptions within organizations. Tarafdar et al. (2015) further revealed that technostress diminishes employees’ innovative behavior and job performance. However, moderate technological challenges may foster learning and adaptation, producing a “double-edged sword” effect.

With the widespread adoption of advanced information technologies, mobile devices, and digital platforms, technostress has become an increasingly salient issue in contemporary organizations. Recent reviews and meta-analytical evidence indicate that technostress is associated with a wide range of negative outcomes, including emotional exhaustion, reduced well-being, impaired performance, and lower work engagement (Bahamondes-Rosado et al., 2023; Kumar, 2024; Nastjuk et al., 2024). As digital work arrangements increasingly blur temporal and spatial boundaries, technostress has been shown to undermine employees’ capacity to sustain positive work-related states, particularly in technology-intensive environments (Korzynski et al., 2021; Tarafdar et al., 2020).

In summary, technostress has evolved from the early notion of “computer anxiety” into a comprehensive reflection of the modern digital workplace, with its definition and implications continuing to expand. It not only reflects employees’ psychological strain in using technology but also profoundly affects their work attitudes, well-being, and organizational behavior.

Building on the evolving understanding of technostress in organizational contexts, the present study situates technostress within the Conservation of Resources (COR) theory to clarify its role in the proposed research model. From this perspective, digital leadership represents a form of organizational support that provides employees with conditional resources, including guidance, structure, and learning opportunities, which help them cope with the uncertainty and demands associated with digital transformation. In contrast, digital self-efficacy functions as a critical personal psychological resource by enhancing employees’ sense of control and confidence in technology rich environments, thereby facilitating resource gain processes.

Technostress, however, reflects a salient contextual demand that may accelerate the depletion of employees’ energy and cognitive resources, thereby triggering potential resource loss processes. When employees experience high levels of technostress, their capacity to transform leadership provided resources into personal efficacy resources is likely to be constrained. Conversely, when organizational conditional resources and individual efficacy resources jointly contribute to resource accumulation, employees are more likely to sustain higher levels of work engagement. Accordingly, the COR theory not only illuminates dynamic processes of resource loss and gain but also provides a coherent theoretical foundation for examining how digital leadership, digital self-efficacy, and technostress jointly shape employee outcomes in digitalized work contexts.

### 2.2 Digital leadership and employee engagement

Digital leadership is defined as a leader’s capability to utilize digital technologies, strategic vision, and empowering behaviors to guide employees in adapting to technological changes and promoting organizational innovation during digital transformation (Zeike et al., 2019). In the present study, digital leadership is conceptualized as a critical contextual resource that shapes employees’ motivational states and work engagement in digital work environments.

Recent empirical studies increasingly demonstrate that digital leadership plays a critical role in fostering employee work engagement in organizations undergoing digital transformation. For example, Li et al. (2024) found that middle managers’ digital leadership significantly enhanced employees’ work engagement by providing technological guidance and empowering support in digital work settings. Similarly, Yang et al. (2024) showed that digital leadership positively influenced employees’ voice behaviors through the mediating role of work engagement, highlighting engagement as a key motivational mechanism through which digital leadership shapes employee outcomes.

More recent research further extends this evidence by examining engagement in technology intensive contexts (Khan et al., 2025) demonstrated that digital leadership promotes employees’ techno work engagement and empowerment in public healthcare organizations undergoing digital transformation. Likewise, Khan et al. (2024) reported that leadership practices supporting digital transformation significantly enhanced employees’ techno work engagement in the public sector. Together, these recent studies provide robust and current empirical evidence that digital leadership constitutes an important antecedent of employee engagement in contemporary digital work environments.

According to the COR theory, employees strive to acquire, preserve, and protect valuable resources, and leaders play an essential role in providing and distributing them (Hobfoll, 1989, 2001). The development of work engagement is shaped by three psychological resource conditions: meaningfulness, safety, and availability. Digital leadership supports these dimensions, thereby enhancing employees’ engagement (Kahn, 1990).

Digital leaders enhance employees’ sense of work meaning and value by articulating the vision and long-term benefits of digital transformation (Salanova and Schaufeli, 2008). By emphasizing creativity and learning within digital work, leaders stimulate employees’ vitality and intrinsic motivation, which enhances the vigor and absorption dimensions of work engagement (Bakker and Demerouti, 2008). Research indicates that employees who perceive their work as meaningful are more inclined to invest emotional and cognitive resources to achieve personal and organizational performance goals (Saks, 2006).

During organizational transformation, digital leaders cultivate open communication, tolerance for errors, and a fast learning culture, which reduce employees’ fear of technological failure and enhance psychological safety (Monje-Amor et al., 2021). Leaders’ emotional support and recognition act as vital affective resources that alleviate uncertainty and stress linked to digital tool adoption and changes in work design (Hakanen et al., 2006). According to COR theory, when employees perceive a low risk of resource loss, they are more likely to invest additional resources in their work to obtain greater returns (Hobfoll, 1989).

Furthermore, digital leadership develops employees’ skills and digital self-efficacy through training, knowledge sharing, and technological support, helping them feel capable and confident, which improves psychological availability (Xanthopoulou et al., 2007). Leaders’ role modeling and technological expertise enhance employees’ self-efficacy through social learning and reduce the resource strain caused by technological change (Brod, 1984; Tarafdar et al., 2007). Research demonstrates that employees with ample resources are more likely to experience focus and absorption, thereby displaying stronger work engagement (Schaufeli et al., 2006).

In summary, digital leadership enhances employees’ sense of meaningfulness by articulating a clear digital vision and aligning technological change with organizational goals. It strengthens psychological safety by promoting open communication, tolerance for errors, and supportive learning environments. In addition, digital leadership increases psychological availability by providing training, structural support, and resources that enhance employees’ confidence and capacity to engage in digital work. When these psychological needs are satisfied, employees are more likely to invest personal resources in their work and organizational objectives. This aligns with the central proposition of COR theory, which suggests that when employees receive additional resources from their organization, they are motivated to invest greater emotional and cognitive energy to achieve further resource gains (Hobfoll, 1989).

*H1*: Digital leadership has a significant positive influence on employee work engagement.

### 2.3 Digital leadership and digital self-efficacy

Self-efficacy, a central concept in Social Cognitive Theory (SCT), refers to an individual’s belief in their ability to organize and perform actions effectively to achieve intended goals (Bandura, 1982, 1997). Within digital transformation, this concept has evolved into digital self-efficacy, which reflects employees’ confidence and perceived competence in using digital technologies to perform work tasks (Tramontano et al., 2021). Empirical research suggests that self-efficacy mainly arises from direct task experience and positive feedback, which enhance individuals’ subsequent confidence and performance (Stumpf et al., 1987). Zhu et al. (2025) demonstrated that digital leadership significantly improves employees’ digital self-efficacy in the hospitality industry by facilitating digital learning, encouraging experimentation, and strengthening confidence in using digital technologies.

Additional studies further support a close association between digital leadership and employees’ digital self-efficacy across different organizational settings. Srivastava et al. (2023) showed that digital transformational leadership enhances individuals’ digital self-efficacy as well as their digital agility in higher education institutions undergoing digital transformation. Moreover, Mamdouh et al. (2025) reported that digital leadership exerts a significant positive effect on employees’ digital self-efficacy, which in turn contributes to improved work motivation and performance in technology intensive organizations. These recent studies provide consistent empirical evidence that digital leadership serves as an important antecedent of employees’ digital self-efficacy across diverse digital transformation contexts.

In organizational settings, digital leadership promotes employees’ digital self-efficacy through technical training, resource provision, and structural support, which foster positive experiences and reduce learning costs (Dery et al., 2017; Zeike et al., 2019). Moreover, based on Social Learning Theory, digital leaders promote vicarious learning by serving as role models and encouraging knowledge sharing among colleagues. Specifically, by demonstrating effective uses of digital tools, sharing best practices, and showcasing successful digital initiatives, leaders allow employees to observe successful experiences of others, thereby strengthening employees’ beliefs in their own digital capabilities (Bandura, 1997; Cortellazzo et al., 2019). Digital leaders exert verbal persuasion by providing encouragement, coaching, and confidence enhancing communication to employees. Specifically, by offering timely feedback, positive reinforcement, and explicit expressions of trust in employees’ digital capabilities, leaders strengthen employees’ beliefs that they can successfully perform digital tasks, thereby further enhancing their digital self-efficacy.

From the COR perspective, digital leadership offers skill, emotional, and structural resources—such as training, supportive feedback, psychological safety, and technological infrastructure—that enable employees to accumulate, maintain, and expand their psychological and capability resources (Dery et al., 2017; Hobfoll, 2001). The accumulation of these resources increases employees’ control and confidence in using technology, thereby reinforcing their digital self-efficacy.

In digital workplaces, leaders who foster a supportive climate, reduce punitive responses to failure, and encourage experimentation can alleviate employees’ technological anxiety and uncertainty, thereby enhancing their digital self-efficacy (Gilson et al., 2015; Hoch and Kozlowski, 2014; Mazmanian et al., 2013). Additionally, by fostering empowerment and trust, digital leaders enhance employees’ autonomy, allowing them to retain confidence and competence even in remote or uncertain contexts (Cortellazzo et al., 2019; Hoch and Kozlowski, 2014).

In summary, digital leadership reinforces employees’ control and confidence by providing diverse resources through skill development, role modeling, structural support, and emotional empowerment. Drawing on an integrated framework of Social Cognitive Theory and the Conservation of Resources theory, this study proposes the following hypothesis:

*H2*: Digital leadership has a significant positive effect on digital self-efficacy.

### 2.4 Digital self-efficacy and work engagement

Recent empirical studies consistently indicate that digital self-efficacy plays an important role in fostering employee work engagement in digital and technology intensive work environments. Digital self-efficacy reflects employees’ confidence in their ability to effectively use digital technologies to accomplish work tasks, which has become increasingly critical in contexts characterized by rapid digitalization, remote collaboration, and continuous technological change.

Growing empirical evidence supports a positive relationship between digital self-efficacy and work engagement. For example, Liu et al. (2025) found that teachers with higher levels of digital self-efficacy exhibited stronger work engagement in online teaching contexts. Similarly, Shi et al. (2025) demonstrated that digital self-efficacy serves as an important psychological mechanism linking digital competence development to work engagement and overall work well-being in post pandemic educational settings. Evidence from digitally mediated work contexts further reinforces this relationship Briones et al. (2023) showed that employees’ e work self-efficacy, a construct conceptually aligned with digital self-efficacy, was positively associated with work engagement among teachers engaged in technology mediated work. Together, these studies provide up to date empirical support for digital self-efficacy as a key antecedent of work engagement in contemporary digital work environments.

According to the COR theory, individuals strive to acquire, preserve, and safeguard valued resources to manage external stressors and reduce potential losses (Hobfoll, 1989). Within this theoretical framework, digital self-efficacy (DSE) is conceptualized as a crucial psychological resource that enables employees to sustain confidence and control amid technological complexity, remote collaboration challenges, and information overload, thereby mitigating resource depletion and enhancing work motivation (Tramontano et al., 2021; Xanthopoulou et al., 2007).

Consistent with COR theory, self-efficacy, as a personal resource, alleviates stress caused by job demands and fosters work engagement by strengthening goal-directed behavior and emotional regulation. Employees who exhibit higher digital self-efficacy therefore tend to display greater vigor, dedication, and absorption in digital work settings, thereby sustaining a prolonged state of engagement (Bakker and Demerouti, 2008; Schaufeli et al., 2006).

Moreover, digital self-efficacy enhances employees’ confidence and adaptability in technology use, allowing them to remain focused and emotionally stable when encountering disruptions, information interruptions, or learning-related stress (Compeau and Higgins, 1995). This process of resource accumulation aligns with the “resource gain spiral” in the Conservation of Resources theory, indicating that individuals who acquire new resources are more inclined to invest additional effort to obtain further gains (Hobfoll, 2001).

Overall, digital self-efficacy fosters sustained work engagement in digital contexts by enhancing employees’ sense of control, psychological security, and positive work experiences. Accordingly, this study proposes the following hypothesis:

*H3*: Digital self-efficacy has a significant positive effect on work engagement.

### 2.5 The mediating role of digital self-efficacy

In digital work environments, digital leadership not only directly influences employees’ attitudes and behaviors, but also indirectly promotes positive work-related outcomes by activating key psychological mechanisms. Recent research increasingly adopts a resource-based perspective to suggest that digital self-efficacy functions as a crucial mediator linking contextual support to employees’ work outcomes.

Recent empirical studies indicate that digital leaders enhance employees’ digital self-efficacy by providing digital training, technological support, and learning opportunities, thereby facilitating positive behavioral and performance outcomes. For example, Zhu et al. (2025) found that digital leadership indirectly promotes employees’ innovative behavior through the enhancement of digital self-efficacy. Sun and Yoon (2025) demonstrated that, in the context of digital transformation in higher education, digital self-efficacy plays a key mediating role between digital transformation initiatives and faculty performance. These findings suggest that employees’ confidence in their digital capabilities serves as an important psychological bridge through which external digital investments are translated into tangible outcomes.

In addition, digital leaders cultivate a learning-oriented culture and empowerment practices that enhance employees’ confidence in engaging with emerging technologies. Such a climate encourages continuous learning, adaptability, and the reinforcement of positive efficacy beliefs (Maran et al., 2022). As employees’ digital self-efficacy improves, they perceive less strain from resource depletion and show greater willingness to explore new technologies, enhance efficiency, and pursue innovation (Rohatgi et al., 2016). Higher levels of self-efficacy reduce employees’ technological anxiety (Porto Bellini et al., 2016) while enhancing their persistence and perceived competence in completing digital tasks (Agarwal et al., 2000; Rohatgi et al., 2016).

Recent research further indicates that digital self-efficacy not only helps alleviate employees perceived stress arising from technological complexity and continuous learning demands but also enhances their persistence and motivational investment in digital tasks (Liu et al., 2025), in an online teaching context, revealed that digital self-efficacy operates as part of a chain mediation mechanism that transforms organizational support into higher levels of work engagement. Likewise, Dong (2025) reported that digital self-efficacy mediates the relationship between digital learning engagement and individual motivation, underscoring its central role in the resource conversion process.

From a Conservation of Resources perspective, digital self-efficacy can be conceptualized as a critical personal psychological resource that enables employees to maintain a sense of control and confidence when facing technological uncertainty and digital challenges. Employees with higher levels of digital self-efficacy are better able to convert external resources provided by digital leaders into internal motivation and sustained work engagement, thereby reducing resource loss and facilitating positive resource gain processes. Based on the above theoretical reasoning and recent empirical evidence, this study proposes that digital self-efficacy serves as a key mediating mechanism between digital leadership and employee work engagement.

*H4*: Digital self-efficacy mediates the relationship between digital leadership and work engagement.

### 2.6 The moderating role of technostress

During digital transformation, employees enjoy the convenience and efficiency brought by digital technologies, yet they inevitably encounter technostress. Technostress is defined as the psychological and physiological strain that arises when employees struggle to adapt to new technologies (Tarafdar et al., 2007). This form of stress generally stems from five key dimensions: overload, complexity, invasion, insecurity, and uncertainty (Ragu-Nathan et al., 2008). Although digital leadership provides employees with valuable resources, such as guidance, support, and learning opportunities, the extent to which these resources are translated into digital self-efficacy may depend on employees’ exposure to technostress.

According to the COR theory, individuals seek to acquire, preserve, and protect valued resources, while the threat of resource loss produces particularly strong psychological effects (Hobfoll, 1989). Within digital environments, technostress acts as a significant resource-depleting factor, consuming employees’ cognitive capacities such as attention and emotional regulation (Ayyagari et al., 2011; Kim et al., 2022). Consequently, employees find it difficult to absorb and internalize the training and support provided by their organizations. When experiencing resource depletion, additional organizational support may not effectively translate into improved digital self-efficacy (Halbesleben et al., 2014; Hobfoll, 2001). Recent research suggests that high levels of technostress impair employees’ ability to effectively utilize available resources, as excessive technological demands consume cognitive and emotional capacity needed for learning and adaptation (Kumar, 2024; Nastjuk et al., 2024). Empirical studies further indicate that technostress weakens positive relationships between supportive organizational practices and desirable employee outcomes, such as engagement and well-being (Harunavamwe et al., 2025; Tarafdar et al., 2020). Under conditions of elevated technostress, employees are more likely to experience fatigue, anxiety, and attentional overload, which may hinder the internalization of leadership-provided resources.

From a resource conversion perspective, digital leadership provides both conditional and emotional resources, yet their conversion depends on employees’ psychological capacity and cognitive bandwidth (Halbesleben et al., 2014). When technostress levels are high, employees’ resources are consumed by coping efforts, thereby diminishing their sensitivity and responsiveness to leadership support. Systematic reviews have shown that leaders’ technical and social support can mitigate technostress and its consequences; however, their effectiveness varies across digital environments and workload levels (Rademaker et al., 2025). From a Conservation of Resources perspective, technostress represents a resource-depleting condition that increases employees’ vulnerability to further resource loss.

Conversely, when technostress remains low, employees experience less resource depletion and are better able to focus on and utilize leadership-provided training, process optimization, and emotional support, thus generating a “resource gain spiral” (Hobfoll, 2001; Hobfoll et al., 2018). Lower levels of technological tension also alleviate feelings of inefficacy and anxiety, fostering a psychologically safe learning environment that enhances digital self-efficacy (Salanova et al., 2013).

From a social learning perspective, digital self-efficacy is shaped not only by mastery experiences but also by vicarious learning and verbal persuasion (Bandura, 1997). However, elevated levels of technostress divert employees’ attention and heighten cognitive load, thereby diminishing their ability to internalize leaders’ digital modeling and verbal persuasion (Kim et al., 2022; Salanova et al., 2013).

Accordingly, when technostress is high, the positive effects of digital leadership on employees’ digital self-efficacy are expected to be attenuated. In contrast, under conditions of low technostress, employees possess greater remaining resources to absorb leadership support, engage in digital learning, and develop confidence in their digital capabilities. Therefore, technostress is expected to function as a negative boundary condition that constrains the conversion of leadership-provided resources into digital self-efficacy.

*H5*: Technostress negatively moderates the relationship between digital leadership and digital self-efficacy. Specifically, when technostress is low, the positive influence of digital leadership on digital self-efficacy becomes stronger, whereas under high technostress, this positive relationship diminishes.

**Figure 1:**
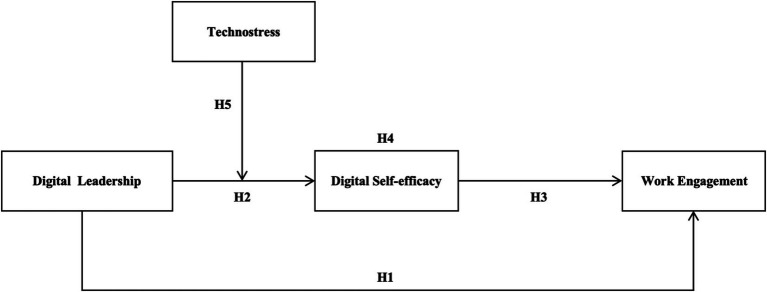
Theoretical model.

Based on the above theoretical background and research hypotheses, a conceptual model is proposed to describe the impact of digital leadership on work engagement, as shown in Figure 1.

## 3 Materials and methods

### 3.1 Sample and procedures

Data for this study were collected from March 2025 to April 2025. The research sample mainly included employees in China. A formal survey was conducted using a web-based questionnaire. Approval was guaranteed by the relevant human resource heads of the companies, who willingly participated in the surveys. This study separated the independent variables from the dependent variables in survey waves to mitigate common method bias (Podsakoff et al., 2012). The questionnaire survey comprised two stages: During Time 1 (T1), employees completed questionnaires regarding a predictor variable (digital leadership), a mediating variable (digital self-efficacy), and demographic variables (age, gender, education and seniority). After a month, during Time 2 (T2), the same participants completed questionnaires regarding a moderating variable (technostress) and a dependent variable (work engagement). This time interval was chosen to maintain sufficient temporal proximity to capture the proposed psychological processes while minimizing respondents’ consistency effects. To match the responses obtained during T1 and T2, participants were asked to enter the last four digits of their phone numbers in the questionnaires. After the matching procedure was completed, all data were anonymized and any potentially identifying information was removed prior to analysis. These procedures were implemented to ensure participants’ confidentiality and to comply with standard ethical guidelines for survey research.

In this study, 560 questionnaires were distributed, and 407 were returned. After removing incomplete responses and outliers, 352 valid questionnaires remained, resulting in a response rate of 62.1%. Regarding gender distribution, 167 respondents were male and 185 were female, representing 47.44 and 52.56% of the total sample, respectively. Most respondents were aged between 26 and 35 years, accounting for 46.31% of the total sample. Most participants held a bachelor’s degree, accounting for 48.01%. In terms of Seniority, 44.03% had between 1 and 3 years of tenure. Additionally, the sample size was sufficient to support the model analysis. According to the rule of thumb that requires a sample size of five times the number of questionnaire items, the minimum requirement was 265 responses based on 53 items (Faul et al., 2009). Therefore, the final sample of 352 respondents exceeded the minimum requirement, ensuring the adequacy of the sample size.

### 3.2 Measures

The scales used in this study were mainly derived from mature scales used in the academic community, with proven reliability and validity in domestic and foreign studies. All scales used the 5-point rating like the Likert scale, where 1 means strongly disagree and 5 means strongly agree. The specific measurement of each variable is as follows.

#### 3.2.1 Digital leadership

Digital leadership was measured with the 6-item scale developed by Zeike et al. (2019). The items are as follow: “my leader think using digital tools is fun,” “my leader is a digital expert,” “When it comes to digital knowledge, my leader is always up to date” etc. Cronbach’s alpha for this scale was 0.871.

#### 3.2.2 Digital self-efficacy

Digital Self-efficacy was measured with the 15-item scale developed by Tramontano et al. (2021), containing 5 dimensions: e-skills, Trust building, Self-care, Remote Social and Remote Emotional. The items are as follows: “Manage your time effectively, even if you have to juggle personal and professional commitments?” “Understand when technology usage is impacting your wellbeing, even if you are very focused on some work tasks?” “Utilize a range of social networking tools to maximize your work relationships?” etc. Cronbach’s alpha for this scale was 0.925.

#### 3.2.3 Work engagement

Work engagement was measured with the 9-item scale developed by Schaufeli et al. (2006), containing 3 dimensions: vigor, dedication and absorption. The items are as follows: “At my work, I feel bursting with energy,” “I am enthusiastic about my job,” “I get carried away when I am working” etc. Cronbach’s alpha for this scale was 0.919.

#### 3.2.4 Technostress

Technostress was measured with the 23-item scale developed by Tarafdar et al. (2007), containing 5 dimensions: Techno overload, Techno invasion, Techno complexity, Techno insecurity, Techno uncertainty. The items are as follows: I am forced by this technology to do more work than I can handle,” “I do not know enough about this technology to handle my job satisfactorily,” “I feel constant threat to my job security due to new technologies” etc. Cronbach’s alpha for this scale was 0.937.

#### 3.2.5 Control variables

To minimize the potential influence of extraneous variables on the examined relationships, control variables included employee gender, age, education level and seniority (Table 1).

**Table 1:** Demographics of the survey respondents (*N* = 352).

Variable	Options	*N*	Percentage
Gender	Male	167	47.44
	Female	185	52.56
Age	≤ 25	110	31.25
	[26, 35]	163	46.31
	[36, 45]	47	13.35
	[46, 55]	20	5.68
	≥ 56	12	3.41
Education	High school	50	14.20
	Associate degree	58	16.48
	Bachelor degree	169	48.01
	Master degree	73	20.74
	doctoral degree	2	0.57
Seniority	≤ 1	24	6.82
	[1, 3]	155	44.03
	[4, 6]	102	28.98
	[7, 10]	48	13.64
	≥ 10	23	6.53

## 4 Results

All statistical analyses were conducted using Mplus 8.0, and SmartPLS 4.1. SEM was run with Mplus 8.0 and SmartPLS 4.1 to test reliability, validity, and model fit. To examine the hypotheses, this study performed partial least squares structural equation modelling (PLS-SEM) using SmartPLS 4.1 software.

### 4.1 Reliability and validity

All first order constructs, including the five dimensions of technostress which are techno overload, techno invasion, techno complexity, techno insecurity and techno uncertainty, were modeled using multiple reflective indicators. Technostress was modeled as a reflective second order construct, with its five dimensions serving as first order constructs. We evaluated construct reliability through internal consistency analysis and obtained acceptable values for both Cronbach’s alpha and composite reliability. However, four items, namely DSE13, TS9, TS18, and TS19, exhibited factor loadings lower than 0.5 within the digital self-efficacy and technostress constructs, which did not meet the recommended threshold according to Hair et al. (2019). As a result, these items were removed to improve the internal consistency and reliability of the model and to prevent possible model fit problems. Table 2 summarizes the results of convergent validity and internal consistency reliability. All indicators and constructs meet the required measurement criteria. Specifically, the factor loadings are all above 0.656, demonstrating that indicator reliability is achieved (Henseler et al., 2009). In addition, Cronbach’s alpha value of each construct ranged from 0.871 to 0.937 (exceeding 0.7). The AVE values ranged from 0.506 to 0.653 (exceeding 0.5), denoting that convergent validity is also achieved. Furthermore, CR values are 0.903 to 0.935, well above the required minimum level of 0.70, thus demonstrating internal consistency (Hair et al., 2014). In other words, the results show that the model has good convergent validity and internal consistency.

**Table 2:** Construct reliability and validity.

Variable	Item**s**	Loading	Cα	CR	AVE
DL	6	0.749–0.843	0.871	0.903	0.608
DSE	14	0.656–0.750	0.925	0.935	0.506
WE	9	0.731–0.850	0.919	0.933	0.607
Second order					
TS	20	0.762–0.845	0.937	0.904	0.653
TS: TO	5	0.792–0.871			
TS: TI	3	0.845–0.873			
TS: TC	5	0.792–0.839			
TS: TIS	3	0.847–0.862			
TS: TU	4	0.810–0.830			

*N* = 352; DL, digital leadership; DSE, digital self-efficacy; WE, work engagement; TS, technostress; TO, techno-overload; TI, techno-invasion; TC, techno-complexity; TIS, techno-insecurity; TU, techno-uncertainty.

For discriminant validity, compared to other competition models, the theoretical four-factor model (digital leadership, digital self-efficacy, work engagement and technostress) had a better fit to the data [*χ*^2^/df = 1.483, (CFI) = 0.943, (TLI) = 0.940, (RMSEA) = 0.037, and (SRMR) = 0.039] (see Table 3). The CFA results showed that the theoretical four-factor model had satisfactory discriminant validity.

**Table 3:** Results of confirmatory factor analysis.

Models	Factor	*χ* ^2^	df	*χ*^2^/df	RMSEA	CFI	TLI	SRMR
Four-factor model	DL, DSE, WE, TS	1655.203	1,116	1.483	0.037	0.943	0.940	0.039
Three-factor model	DL + DSE, WE, TS	2187.868	1,119	1.955	0.052	0.887	0.881	0.056
Two-factor model	DL + DSE + WE, TS	2831.544	1,121	2.526	0.066	0.819	0.810	0.066
Single-factor model	DL + DSE + WE+TS	5170.702	1,127	4.588	0.101	0.572	0.554	0.109

Furthermore, the Heterotrait–Monotrait ratio of correlations (HTMT) was used to evaluate discriminant validity. According to the conservative criterion, HTMT values should remain below 0.85 (Tabri and Elliott, 2012). As presented in Table 4, all HTMT values fall between 0.190 and 0.738, remaining below the conservative benchmark, thus confirming discriminant validity.

**Table 4:** Heterotrait–Monotrait ratio.

Variable	DL	DSE	TS					WE
			TSA	TSB	TSC	TSD	TSE	
DL								
DSE	0.597							
TS	0.276	0.586	n.a.					0.451
TSA	0.259	0.513						
TSB	0.261	0.501	0.738					
TSC	0.219	0.502	0.643	0.670				
TSD	0.190	0.443	0.647	0.692	0.658			
TSE	0.240	0.521	0.684	0.686	0.672	0.648		
WE	0.628	0.662	0.418	0.402	0.394	0.334	0.354	

### 4.2 Common method variance

Common method variance (CMV) may affect the empirical results because our study data were collected through self-report questionnaires. Podsakoff et al. (2003) showed that procedural and statistical techniques can be adopted for CMV. In the statistical technique, the possibility of common method bias was tested using Harman’s one factor test (Podsakoff and Organ, 1986). A principal component factor analysis with varimax rotation was used on the items of digital leadership, digital self-efficacy, work engagement and technostress. This result revealed multiple factors with eigenvalues greater than 1. The first factor accounted for 13.37% (< 50%) loading, which proved the absence of CMV (Woszczynski and Whitman, 2004).

Further, we conducted the unmeasured latent method factor (Podsakoff et al., 2003), to test CMV. A comparison of the latent method factor model (*χ*^2^/df = 1.483, CFI = 0.952, TLI = 0.947, RMSEA = 0.035, SRMR = 0.044) and the four-factor model (*χ*^2^/df = 1.424, CFI = 0.943, TLI = 0.940, RMSEA = 0.037, SRMR = 0.039) indicated the change in CFI did not exceed the recommended cutoff of 0.01 (Cheung and Rensvold, 2002). Thus, CMV was not a major problem for the data.

### 4.3 Means and correlations

Table 5 presents the means, standard deviations, and correlations among the study variables. Digital leadership is positively correlated to digital self-efficacy (r = 0.536**), and work engagement (r = 0.561**). Digital self-efficacy is positively correlated to work engagement (r = 0.609**). Technostress is negatively correlated to digital leadership (r = −0.250**), digital self-efficacy (r = −0.544**), and work engagement (r = −0.419**).

**Table 5:** Means, standard deviations, and correlations among the study variables.

Variables	Mean	SD	1	2	3	4	5	6	7	8
1. Gender	1.53	0.500	1							
2. Age	2.04	0.991	−0.080	1						
3. Education	2.77	0.955	0.009	−0.530**	1					
4. Seniority	2.69	1.009	−0.123*	0.693**	−0.402**	1				
5. DL	3.75	0.826	0.035	0.005	0.085	−0.068	1			
6. WE	3.80	0.790	−0.026	−0.225**	0.287**	−0.295**	0.561**	1		
7. DSE	3.66	0.796	−0.015	−0.189**	0.250**	−0.211**	0.536**	0.609**	1	
8. TS	3.22	0.834	0.033	0.084	−0.119*	0.129*	−0.250**	−0.419**	−0.544**	1

*N* = 352; **p < 0.01; *p < 0.05.

### 4.4 Structural model

Before testing the structural model, we evaluated the presence of multicollinearity among the constructs. Multicollinearity was assessed using the variance inflation factor (VIF), and, ideally, the VIF values should be close to and lower than 3 (Hair et al., 2019). The results indicated that all variance inflation factor values were below the threshold, with the maximum value being 2.595, suggesting the absence of significant multicollinearity among the constructs. We also examined the R^2^ value, which indicates the model’s predictive power by showing the endogenous variable’s variance that the exogenous variables can explain. The R^2^ value for WE (0.499) indicate that all the constructs combined explain 49.9% of the variance in WE. The R^2^ value for DSE is 0.506. Further, we checked the Q^2^ values to assess the predictive relevance values generated by the variables. The Q^2^ values for WE (0.442) and DSE (0.492) were above 0, which means that the model has predictive relevance.

To examine the hypotheses, bootstrapping was carried out using SmartPLS 4.1 with 5,000 subsamples based upon percentile bootstrapping with a two-tailed test type and a significance level of 0.05. The PLS-SEM bootstrapping approach statistically determined the structural mode coefficients representing the hypothesized relationships.

### 4.5 Direct effect and mediation effect testing

**Figure 2:**
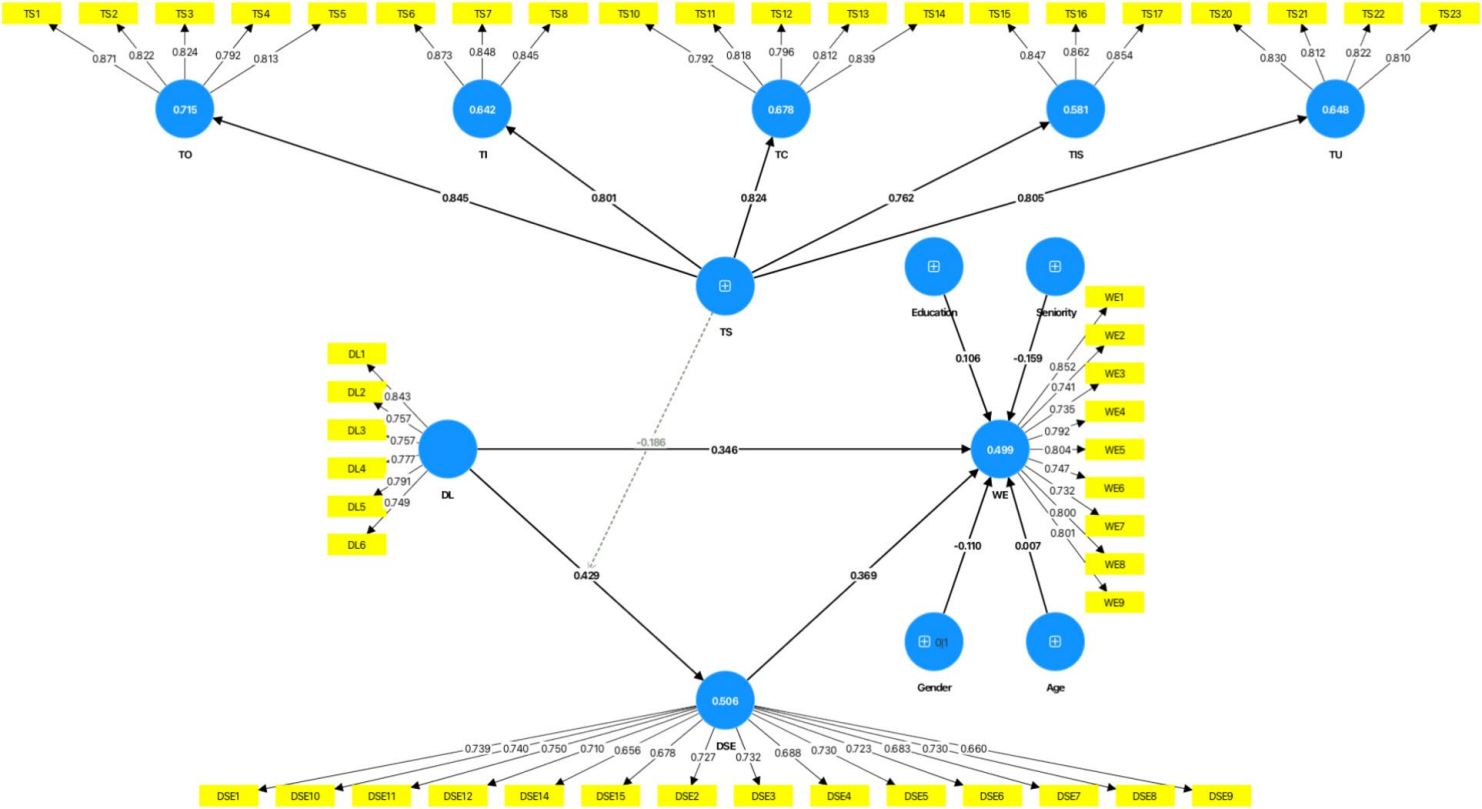
PLS path model from Smart PLS.

Figure 2 and Table 6 portray the results of the structural path analysis. The results show that digital leadership has a significant positive impact on work engagement (B = 0.346; *p* < 0.001; 95% CI: 0.250, 0.439), supporting Hypothesis 1. Digital leadership has a significant positive impact on digital self-efficacy (B = 0.429; *p* < 0.001; 95% CI: 0.349, 0.512), supporting Hypothesis 2. And digital self-efficacy has a significant positive impact on work engagement (B = 0.369, *p* < 0.001, 95% CI: 0.283, 0.459), supporting Hypothesis 3.

**Table 6:** Hypothesis testing.

Hypotheses	Relationship	B	Std. dev	T statistics	*P*-value	LLCI	ULCI	Results
Direct effect								
H1	DL → WE	0.346	0.049	7.105	0.000	0.250	0.439	Supported
H2	DL → DSE	0.429	0.041	10.507	0.000	0.349	0.512	Supported
H3	DSE → WE	0.369	0.045	8.186	0.000	0.283	0.459	Supported
Mediation effect								
H4	DL → DSE → WE	0.158	0.025	6.346	0.000	0.114	0.212	Supported
Moderation effect								
H5	TS × DL → DSE	−0.186	0.036	5.126	0.000	−0.257	−0.113	Supported

Further, the results show that digital self-efficacy significantly mediates the relationship between digital leadership and work engagement (B = 0.158, *p* < 0.001, 95% CI: 0.114, 0.212). Therefore, Hypotheses 4 is supported.

### 4.6 Moderating effect testing

**Figure 3:**
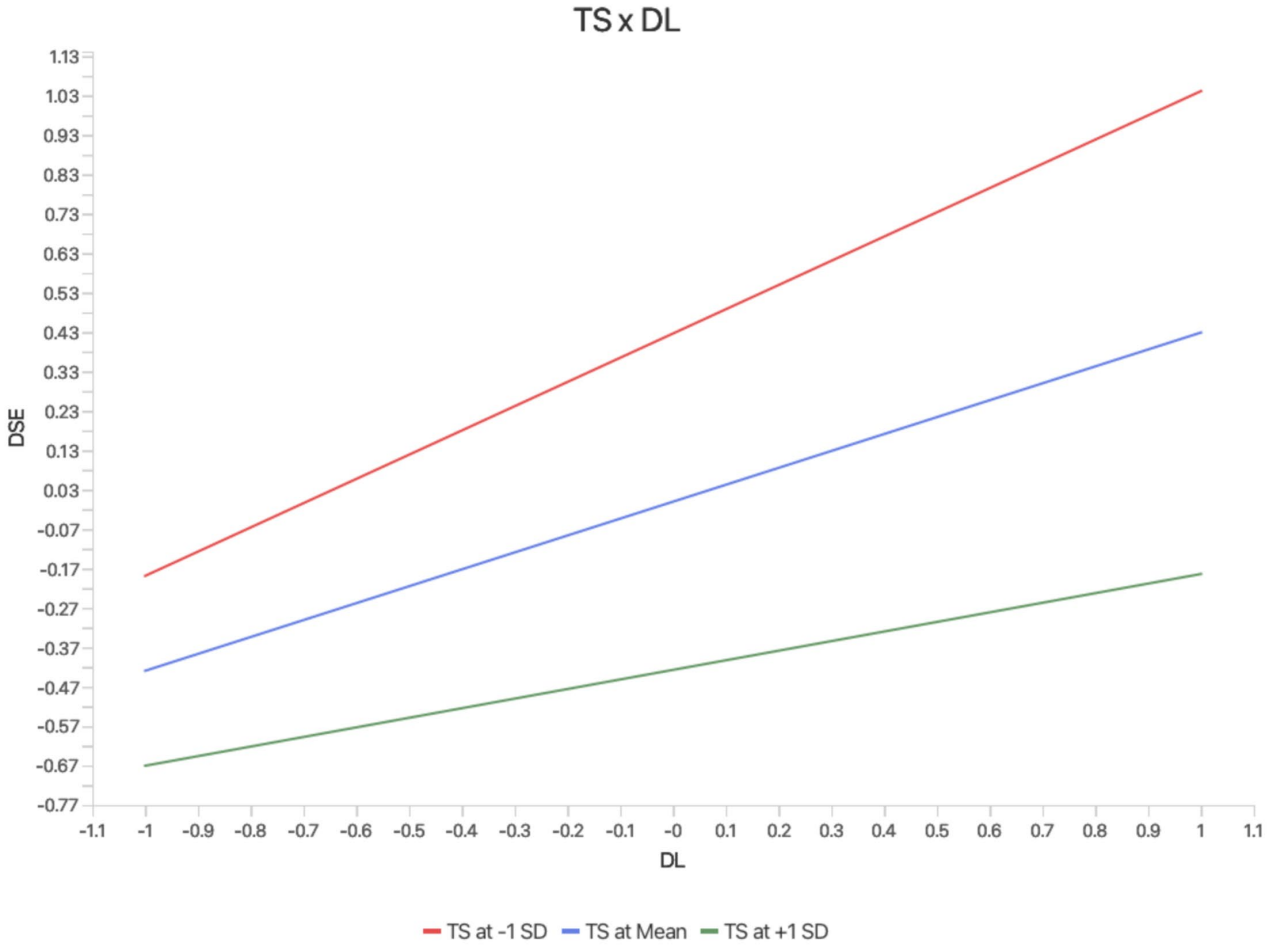
Moderating effect of technostress on the relationship between digital leadership and digital self-efficacy.

This study examined whether information technology stress moderates the relationship between digital leadership and digital self-efficacy. As shown in Figure 3 and Table 6, the interaction between digital leadership and technostress has a significant negative impact on digital self-efficacy (B = −0.186; *p* < 0.001; 95% CI: −0.257, −0.113). This finding suggests that technostress weakens the positive effect of digital leadership on digital self-efficacy, supporting Hypothesis 5. Following (Toothaker, 1994), we plotted simple slope graphs to illustrate the relationship between digital leadership and digital self-efficacy at three different levels: one standard deviation below the mean, equal to the mean, and one standard deviation above the mean. The positive relationship between digital leadership and digital self-efficacy is weaker under high technostress and stronger under low technostress, further confirming the negative moderating effect of technostress.

### 4.7 Moderated mediation testing

To assess the moderated mediation effect, we examined whether the indirect influence of digital leadership on work engagement through digital self-efficacy differed across varying levels of technostress. The findings indicated that the strength of the mediating pathway depended on the level of technostress (see Table 7).

**Table 7:** Results of moderated mediation effect.

Path	Technostress	B	Std. dev	T Statistics	*P*-value	LLCI	ULCI
DL → DSE → WE	High(+1SD)	0.084	0.025	3.334	0.001	0.038	0.139
	Middle	0.148	0.024	6.124	0.000	0.103	0.198
	Low(-1SD)	0.211	0.030	7.002	0.000	0.153	0.273
Index of conditional mediation		−0.076	0.017	4.557	0.000	−0.110	−0.045

At low levels of technostress (−1 SD), the indirect effect of digital leadership on work engagement via digital self-efficacy was the strongest (B = 0.211; *p* < 0.001; 95% CI: 0.153, 0.273). At high levels of technostress (+1 SD), the effect was noticeably weaker (B = 0.084; *p* < 0.001; 95% CI: 0.038, 0.139). Furthermore, the index of moderated mediation (Hayes, 2015) was negative and statistically significant (B = −0.076; *p* < 0.001; 95% CI: −0.110, −0.045), indicating that the indirect effect decreases as technostress increases. These results suggest that higher levels of technostress weaken the mediating role of digital self-efficacy in the relationship between digital leadership and work engagement.

## 5 Discussion and conclusion

This study focuses on digital leadership, digital self-efficacy, technostress, and employee work engagement as its core variables and systematically tests five hypotheses. The empirical results strongly support all hypotheses, demonstrating that this study contributes meaningfully to both theoretical development and practical application.

First, digital leadership exerts a significant positive influence on employees’ work engagement. This finding suggests that within the context of digital transformation, leaders enhance employees’ engagement—encompassing vigor, dedication, and absorption—by promoting digital tools, conducting training, optimizing processes, and empowering employees (Bakker and Demerouti, 2008; Schaufeli et al., 2006).

Second, digital leadership significantly improves employees’ digital self-efficacy, indicating that leaders enhance employees’ confidence and perceived competence in utilizing digital technologies through effective resource provision and supportive mechanisms (Bandura, 1977; Zeike et al., 2019).

Third, digital self-efficacy significantly predicts work engagement, implying that as a key psychological resource, it directly promotes greater vigor, dedication, and absorption among employees (Schaufeli et al., 2006; Xanthopoulou et al., 2007).

Fourth, the mediation analysis shows that digital self-efficacy serves as a partial mediator between digital leadership and work engagement. This suggests that digital leadership not only directly influences engagement but also indirectly enhances it through the reinforcement of employees’ psychological resources.

Finally, the moderation analysis reveals that technostress significantly moderates the relationship between digital leadership and digital self-efficacy. When technostress levels are low, the positive impact of digital leadership on self-efficacy is amplified; conversely, under high technostress, this facilitative effect diminishes substantially (Kim et al., 2022; Rademaker et al., 2025; Ragu-Nathan et al., 2008).

From the perspective of the Conservation of Resources theory, the findings support the dual mechanisms of the “resource gain spiral” and the “resource loss spiral.” Digital leadership provides essential resources that allow employees to build psychological capital in the form of digital self-efficacy, which subsequently enhances their work engagement. However, when technostress is high, accelerated resource depletion hinders employees from transforming leadership-provided resources into personal ones, thereby diminishing the overall effectiveness of digital leadership (Hobfoll, 1989; Hobfoll et al., 2018).

## 6 Theoretical contributions

Grounded in the interaction among digital leadership, digital self-efficacy, and technostress, this study makes substantial contributions at multiple theoretical levels to the existing body of literature.

First, this study deepens and broadens the application of the Conservation of Resources (COR) theory in the context of digital transformation. The findings indicate that digital leadership, as a primary source of organizational resources, directly enhances employees’ work engagement and simultaneously establishes a pathway through which organizational resources are converted into personal resources and, ultimately, into work outcomes by improving employees’ digital self-efficacy. This provides empirical evidence for a cross-level mechanism of resource transmission and addresses a theoretical gap concerning how resources circulate and transform within digital organizations.

Second, integrating self-efficacy theory (Bandura, 1977) into the digital context, this study identifies digital self-efficacy as a central mediating variable. Previous studies have primarily examined general self-efficacy or psychological capital as predictors of work-related outcomes (Xanthopoulou et al., 2007). By contextualizing this construct as employees’ confidence in their ability to effectively use digital technologies (Zeike et al., 2019), the study confirms its partial mediating role between digital leadership and work engagement. This finding elucidates the psychological pathway through which digital leadership shapes employees’ cognitive and emotional states, thereby extending the scope of leadership theory in digital environments.

More importantly, this study identifies technostress as a contextual boundary condition moderating the effects of digital leadership, offering a resource depletion perspective to explain variations in leadership effectiveness. Prior research has demonstrated that technostress stems from system complexity, information overload, and role ambiguity, leading to psychological exhaustion and diminished work efficiency (Ragu-Nathan et al., 2008). However, few studies have explored how technostress affects leadership mechanisms. The findings indicate that under low technostress, resources provided by leaders, including training, technical support, and empowerment, are more readily converted into employees’ digital self-efficacy. Conversely, under high technostress, employees’ attentional resources are drained by information fatigue and cognitive overload, which diminishes their ability to absorb and utilize leadership-provided resources, thereby weakening the positive influence of leadership (Kim et al., 2022; Rademaker et al., 2025).

This suggests that technostress is not only a source of negative emotion but also a crucial moderating factor shaping the efficiency of resource transformation, aligning closely with Hobfoll’s “resource loss spiral” concept (Hobfoll, 1989; Hobfoll et al., 2018).

In summary, this study integrates digital leadership, digital self-efficacy, and technostress into a unified analytical framework, thereby enriching leadership theory in the digital transformation era and providing a novel lens for understanding when and why leadership succeeds or fails. In high technological demand and resource-constrained environments, the effectiveness of leadership becomes dynamic, influenced by employees’ resource conditions and degrees of technostress. These insights lay a theoretical foundation for future research and offer actionable implications for organizations aiming to strengthen employee vitality and engagement in the course of digital transformation.

## 7 Practical implications

This study provides not only theoretical innovation but also actionable insights for leadership practices in digital transformation. The findings indicate that digital leadership plays a pivotal role in enhancing employee engagement (Bakker and Demerouti, 2008). Leaders should integrate technological support, empowerment, emotional consideration, and structural alignment into management practices. Effective digital leaders serve not merely as “technology managers” but as “resource enablers,” offering digital tools and training opportunities to reduce uncertainty and anxiety during technology adoption. Moreover, organizations should embed systematic digital training within their strategic frameworks. Beyond basic skill training, organizations should introduce cross-departmental learning mechanisms, mentoring systems, and digital project simulations that allow employees to acquire practical experience and transform it into digital confidence and sustained engagement. Leaders can leverage learning management systems (LMS) and virtual collaboration platforms to monitor and evaluate training outcomes, thus forming a sustainable learning loop (Zeike et al., 2019). This leader-centered, training-driven resource integration mechanism enables employees to convert organizational support into intrinsic motivation, thereby fostering innovation and performance improvement.

The study further highlights that systematically strengthening employees’ digital self-efficacy is a vital strategy for organizations to adapt to digital transformation and sustain competitiveness. Organizations should establish a multi-level digital empowerment system encompassing: (1) regular digital skill training and coaching; (2) project-based and experiential learning activities, such as digital project simulations; and (3) peer learning and feedback mechanisms that enhance employees’ sense of control and growth in digital contexts. Through these mechanisms, organizations can effectively foster employees’ learning initiative and technological confidence, enabling them to sustain psychological vitality and engagement despite the pressures and uncertainties of digital transformation (Zeike et al., 2019).

This study identifies technostress as a boundary condition that moderates the relationship between digital leadership effectiveness and employee resource development, offering critical managerial insights. Technostress often stems from system complexity, information overload, role ambiguity, and blurred boundaries between work and life, which may result in resource depletion, psychological fatigue, and resistance to innovation (Ragu-Nathan et al., 2008). Therefore, organizations should integrate technostress management into their digital strategies to ensure sustainable transformation and employee well-being. In practice, organizations should assess employees’ technological load and adaptability before implementing new technologies or platforms, adopting phased implementation, real-time support, and responsive feedback systems to prevent resource depletion spirals (Hobfoll, 1989). Managers should also implement clear digital boundary policies, such as restricting communication beyond working hours, promoting flexible scheduling, and formalizing remote work guidelines to mitigate emotional exhaustion arising from the always-on culture. At the organizational level, leadership and HR teams should collaboratively develop Digital Well-being Programs that include counseling, technological load reduction, and employee feedback mechanisms to strengthen organizational resilience and satisfaction. Additionally, management can appoint a Chief Digital Experience Officer (CDEO) or a dedicated task force to continuously monitor employees’ technological satisfaction and stress levels, achieving a dynamic balance between technological advancement and employee welfare.

Finally, organizations should implement ongoing evaluation and feedback systems to dynamically monitor employees’ digital self-efficacy and work engagement, supporting data-driven optimization of resource allocation and leadership strategies. Using standardized tools such as the UWES and the Digital Self-Efficacy Scale, regular assessments can identify high-stress or low-engagement groups and generate empirical insights for refining training systems and leadership approaches.

## 8 Limitations and future research

This study gathered data across multiple time points to reduce common method bias (CMV) and better capture potential causal relationships among variables over time. However, because all variables were based on employees’ self-reports, the potential influence of CMV cannot be fully eliminated. Future research should adopt multi-wave and multi-source data collection approaches to address this concern more rigorously. Moreover, more rigorous experimental designs, such as paired samples, longitudinal tracking, and controlled experiments, could enhance the internal validity and causal inference of future research.

The study sample was primarily drawn from collectivist East Asian regions, which may limit the generalizability of the findings. Because digital transformation differs across countries and industries in technological systems, labor relations, and management logic (Dery et al., 2017), future studies should test this model across diverse national and industrial contexts to explore how cultural values and institutional conditions influence the relationships among digital leadership, technostress, and employee resource responses. For example, in highly regulated or safety-critical organizations, employees’ increased sensitivity to technological errors and data security could diminish the positive effect of digital self-efficacy on work engagement or even evoke defensive psychological reactions.

Although technostress was examined as a single moderating variable, it is inherently multidimensional, including overload, invasion, and complexity, each potentially exerting unique effects on resource transmission (Tarafdar et al., 2007). Future studies should investigate whether distinct dimensions of technostress differentially moderate the relationship between digital leadership and digital self-efficacy (Ragu-Nathan et al., 2008). Furthermore, technostress may not always have negative consequences. A moderate level of stress could stimulate learning motivation and innovation, resulting in an inverted U-shaped relationship. This aligns with the “stress-induced resource investment mechanism” proposed in COR theory (Hobfoll et al., 2018), which merits further empirical exploration in digital environments.

Future research should explore how individual differences serve as boundary conditions that influence the proposed relationships. For example, factors such as digital anxiety, learning orientation, and proactive personality may strongly shape how employees interpret leadership behaviors, perceive resources, and cope with technostress (Srivastava et al., 2015). In addition, organizational factors such as cultural support, IT infrastructure maturity, and digital learning climate may influence how efficiently resources are transmitted across organizational levels (Salanova and Schaufeli, 2008). Furthermore, this study did not explicitly control for industry type or organizational size, both of which may influence the intensity of digital transformation, technological complexity, and resource distribution. Future research is encouraged to incorporate these structural characteristics as control variables or higher-level contextual factors in order to enhance the robustness and generalizability of the findings.

## Data Availability

The raw data supporting the conclusions of this article will be made available by the authors, without undue reservation.
